# Whole-body magnetic resonance imaging in paediatric Hodgkin lymphoma — evaluation of quantitative magnetic resonance metrics for nodal staging

**DOI:** 10.1007/s00247-019-04463-9

**Published:** 2019-07-22

**Authors:** Arash Latifoltojar, Paul D. Humphries, Leon J. Menezes, Athar Haroon, Stephen Daw, Ananth Shankar, Shonit Punwani

**Affiliations:** 1 Centre for Medical Imaging, University College London, 2nd floor, Charles Bell House, 43-45 Foley St., London, W1W 7TS UK; 2 Department of Radiology, University College London Hospitals, London, UK; 3 Institute of Nuclear Medicine, University College London Hospitals, London, UK; 4 Department of Paediatric Haemato-oncology, University College London Hospitals, London, UK

**Keywords:** Adolescents, Children, Diffusion-weighted imaging, Hodgkin lymphoma, Magnetic resonance imaging, Staging

## Abstract

**Background:**

Whole-body MRI is used for staging paediatric Hodgkin lymphoma, commonly using size thresholds, which fail to detect disease in normal-size lymph nodes.

**Objective:**

To investigate quantitative whole-body MRI metrics for nodal characterisation.

**Materials and methods:**

Thirty-seven children with Hodgkin lymphoma underwent 1.5-tesla (T) whole-body MRI using short tau inversion recovery (STIR) half-Fourier-acquisition single-shot turbo-spin-echo and diffusion-weighted imaging (DWI). ^18^Flourine-2-fluoro-2-deoxyglucose (FDG) positron emission tomography (PET)/CT was acquired as the reference standard. Two independent readers assessed 11 nodal sites. The readers measured short-axis-diameter, apparent diffusion coefficient, (ADC) and normalised T2-signal intensity of the largest lymph node at each site. We used receiver operating characteristics (ROC)/area-under-the-curve (AUC) analysis for each MRI metric and derived sensitivity and specificity for nodes with short-axis diameter ≥10 mm. Sub-analysis of sensitivity and specificity was performed with application of ADC cut-off values (<0.77, <1.15 and <1.79×10^−3^ mm^2^ s^−1^) to 5- to 9-mm nodes.

**Results:**

ROC/AUC values for reader 1/reader 2 were 0.80/0.80 and 0.81/0.81 for short-axis-diameter measured using DWI and STIR half-Fourier-acquisition single-shot turbo spin echo, respectively; 0.67/0.72 for normalised T2 signal intensity and 0.74/0.67 for ADC. Sensitivity and specificity for a short-axis diameter ≥10 mm were 84.2% and 66.7% for Reader 1 and 82.9% and 68.9% for Reader 2. Applying a short-axis-diameter ≥10-mm threshold followed by ADC cut-offs to normal-size 5- to 9-mm nodes resulted in sensitivity and specificity for Reader 1 of 88.8% and 60%, 92.1% and 56.7%, and 100% and 16.7%; and for Reader 2, 86.1% and 67.2%, 95.3% and 65.6%, and 100% and 19.7%; and ADC thresholds of <0.77, <1.15, and <1.79×10^−3^ mm^2^ s^−1^, respectively.

**Conclusion:**

Nodal size measurement provides the best single classifier for nodal disease status in paediatric Hodgkin lymphoma. Combined short-axis diameter and ADC thresholds marginally improve sensitivity and drop specificity compared with size classification alone.

## Introduction

^18^Flourine-2-fluoro-2-deoxyglucose (FDG) positron emission tomography (PET) fused with CT scan (FDG PET/CT), performed on a hybrid system, has become the gold standard modality for evaluating lymphoma including Hodgkin lymphoma, with proven improvement at staging and follow-up monitoring compared with either CT or FDG PET alone [1, 2]. However, FDG PET/CT imparts a considerable dose of ionising radiation, which is of particular concern within the paediatric population [3]. Even with low-dose protocols, paediatric patients undergoing a single FDG PET/CT examination are exposed to ionising radiation on the order of 10–20 mSv [4], equivalent to 700–750 chest radiographs. Compared with an adult older than 30 years, exposure to ionising radiation in early childhood causes roughly a tripling in lifetime cancer risk by comparison [5]. Children are inherently more radiosensitive than adults and exposure to ionising radiation is more likely to increase the risk of secondary malignancies (radiation-induced malignancies) in children because of their smaller body size, repeated studies to assess treatment response or relapse, and longer survival following treatment [6, 7]. In paediatric and young adults with Hodgkin lymphoma, more than 90% of patients survive 10 years [8], and hence radiation-induced mortality and risk of developing secondary cancers are of more concern in children and adolescents with Hodgkin lymphoma. Whole-body MRI provides a radiation-free alternative for the staging of paediatric lymphoma [9, 10]. Yet, similar to CT, nodal classification remains dependent predominantly on size measurement, with image contrast used to help localise nodes [11]. Diffusion-weighted imaging (DWI)-derived apparent diffusion coefficient (ADC) has been reported as a potential imaging biomarker for evaluating treatment response in lymphoma [12, 13]. Several studies have suggested that ADC values for malignant lymph nodes are lower compared with benign lymph nodes [14, 15]. However, little has been reported on combining the quantitative parameters provided by sequences such as DWI with standard size measurement to determine whether this provides improved diagnostic accuracy for nodal disease classification [16]. The aim of this study was to determine the potential added diagnostic value of quantitative parameters derived from whole-body MRI for classifying lymph nodes in children and adolescents with Hodgkin lymphoma compared to the FDG PET/CT reference standard.

## Materials and methods

Our institutional review board approved the study and waived the requirement for individual patient consent for use of anonymous clinical and research patient datasets (R&D No: 12/0195 date:16/06/2012).

### Data collection

We reviewed our institutional database for patients referred with suspected Hodgkin lymphoma to the paediatric haemato-oncology clinic. The period between 2009 and 2012 (during which whole-body MRI was part of standard clinical practice at our institution) was interrogated using the following inclusion criteria: (1) confirmed Hodgkin lymphoma on biopsy, (2) age ≤18 years, (3) full whole-body MRI dataset at baseline (below), and (4) FDG PET/CT performed contemporaneously to whole-body MRI. Exclusion criteria were: (1) prior treatment for malignancy other than Hodgkin lymphoma, (2) restaging as part of relapsed Hodgkin lymphoma assessment and (3) incomplete whole-body MRI dataset. Patients were followed with both whole-body MRI and FDG PET/CT for a minimum of 2 years following completion of chemotherapy.

### Whole-body magnetic resonance imaging protocol

Imaging was performed on a 1.5-tesla (T) scanner (Avanto; Siemens, Erlangen, Germany), with the manufacturers’ body- and spine-array coils and the patient in supine position. Immediately prior to imaging, 0.3 mg/kg of body weight of intravenous hyoscine butylbromide (Buscopan; Boehringer Ingelheim, Ingelheim, Germany) was administered. The complete whole-body MRI study comprised respiratory and electrocardiographically gated axial and coronal whole-body short tau inversion recovery (STIR) half-Fourier acquisition single-shot turbo spin echo and whole-body DWI of neck, chest, abdomen and pelvis, complemented by multi-phase dynamic contrast-enhanced imaging of the liver and spleen and breath-held periodically rotated overlapping parallel lines with enhanced reconstruction imaging of the lungs and mediastinum. Diffusion-weighted imaging was performed in the axial plane during free breathing using a STIR echoplanar imaging sequence with diffusion gradients applied in three orthogonal directions at b values of 0 s/mm^2^, 300 s/mm^2^ and 500 s/mm^2^ and calculation of trace-weighted images. A single dose (0.1 mmol/kg) of intravenous gadoterate meglumine (Dotarem; Laboratoire Guerbet, Aulnay-sous-Bois, France) was injected at 3 mL/s, followed by 10 mL of saline flush. At the start of injection, the child was requested to breath-hold for 30 s during rapid dynamic T1-weighted MRI using a 3-D fast low-angle-shot technique. Eighty contiguous 2.5-mm-thick axial images covering the entire liver and spleen were acquired every 5 s. Imaging was then paused for 10 s to allow controlled (instructed) breathing, then two further 5-s acquisitions were performed during a 10-s breath hold, followed by a further 10-s pause for breathing. Interleaved imaging and breath holds were continued for a total of 2 min from the start of intravenous injection. Finally, a multi-breath-holds periodically rotated overlapping parallel lines with enhanced reconstruction MRI was used to acquire images at maximum inspiration to separate the mediastinum from the chest wall and to provide dedicated lung imaging. Twenty-three axial images were acquired with a 3-mm slice thickness during each breath hold. Stacks were repeated to cover the entire chest. The entire protocol was completed in an average of an hour. Full sequence parameters are detailed in Table 1.

**Table 1:** Whole-body magnetic resonance imaging parameters

Parameters	Axial STIR half-Fourier-acquisition single-shot turbo spin echo	Coronal STIR half-Fourier-acquisition single-shot turbo spin echo	Axial STIR diffusion-weighted imaging	Axial T2 periodically rotated overlapping parallel lines with enhanced reconstruction	Axial 3-D fast low-angle-shot for dynamic contrast-enhanced imaging
Repetition time/echo time (ms)	800/60	800/60	4,900/66	3,000/133	2.87/0.93
Inversion time (ms)	130	130	180	N/A	N/A
Matrix	256×256	256×256	128×96	256×256	256×176
Slice thickness (mm)	7	7	4	3	2.5
No. of slices	19	27	27	23	80
Stacks	6–8	2	6–8	2–4	1
Averages	2	2	8	1	1
Echo train	256	256	1	50	1
Flip angle (degrees)	180	180	90	150	9
Parallel acquisition	2	2	2	1	2

*STIR* short tau inversion recovery

### ^18^Flourine-2-fluoro-2-deoxyglucose (FDG) positron emission tomography (PET)/CT protocol

FDG PET/CT scans were acquired using a dedicated combined PET/CT in-line system (Discovery LS; GE Healthcare, Milwaukee, WI). Patients fasted for 6 h prior to scanning, and blood glucose levels were checked to exclude hyperglycaemia (>150 mg/dL). For paediatric patients, the doses were adjusted according to the European Association of Nuclear Medicine paediatric dosage card [17]. ^18^Flourine -FDG (14–370 MBq) was intravenously injected 60 min before imaging. Prior to acquiring the whole-body PET 3-D emission scan, a non-contrast CT was obtained for attenuation correction (80–120 kVp, modulated mA [10–200 mA], pitch 1.375, 3.75-mm slice thickness). Images were acquired at 3 min per bed position as per departmental paediatric protocol.

### ^18^Flourine-2-fluoro-2-deoxyglucose (FDG) positron emission tomography (PET)/CT image analysis

FDG PET/CT images were assessed in consensus by two radionuclide radiologists (L.J.M. and A.H., with 7 and 5 years of experience, respectively). All images were assessed using advanced PET/CT review software (Advantage, version 4.2; GE Medical Systems) with inbuilt capability to scroll through the corresponding PET, CT and fused images in transverse, coronal and sagittal planes and the maximum-intensity projection images. Disease positivity within lymph nodes was defined as the presence of FDG uptake greater than that of the background physiological activity in a clinically suspicious node, or CT short-axis diameter ≥10 mm. The FDG PET/CT positivity/negativity was used as the reference standard against which whole-body MRI was assessed.

### Whole-body magnetic resonance imaging analysis

Whole-body MRI datasets were analysed using Osirix (version 4.1; Pixmeo SARL, Bernex, Switzerland) on a Mac (Apple, Cupertino, CA) workstation. Two radiologists (S.P. and P.D.H., each with more than 10 years of experience with MRI) independently reviewed the whole-body MRI studies blinded to clinical data and FDG PET/CT results. Nodal involvement was assessed in 11 nodal stations (i.e. cervical, supraclavicular, sub-pectoral, axillary, mediastinal, splenic hilar, liver hilar, mesenteric, retroperitoneal, iliac and inguinal regions). The maximum short-axis diameter of the largest nodal mass in a given region on axial STIR half-Fourier-acquisition single-shot turbo-spin-echo and DWI whole-body MRI was measured using software calipers. Disease positivity was defined as a nodal mass with short-axis diameter ≥10 mm. Each of the two original reporting radiologists independently extracted quantitative parameters from the whole-body MRI dataset. Each radiologist selected and marked the largest lymph node (short-axis diameter ≥5 mm) in each nodal station, and recorded the measured size on both axial STIR half-Fourier-acquisition single-shot turbo-spin-echo and b=500 s/mm^2^ diffusion-weighted imaging. We excluded from analysis sites where all nodes were <5 mm in short-axis diameter, to minimise partial volume errors effecting extracted quantitative metrics. For ADC extraction: a region of interest was manually contoured on b=500 s/mm^2^ images and then copied and pasted to subsequent b=300 s/mm^2^ and b=0 s/mm^2^ images. The average signal intensity of the regions of interest (size range 29–763 mm^2^) was calculated at each b value and ADC derived by least squares fit to the signal intensity versus b value plotted [12]. For normalised T2 signal intensity extraction: a region of interest was carefully contoured on axial STIR half-Fourier-acquisition single-shot turbo-spin-echo images and the average region-of-interest signal intensity (size range 22–812 mm^2^) was normalised against right obturator internus muscle signal intensity [18].

### ^18^Flourine-2-fluoro-2-deoxyglucose (FDG) positron emission tomography (PET)/CT and whole-body MRI anatomical matching

Anonymised whole-body MRI and FDG PET/CT datasets were assessed in consensus by two reporting radiologists, a radionuclide radiologist and a paediatric haemato-oncologist. Lymph nodes were visually matched between whole-body MRI and FDG PET/CT, with the node selected for whole-body MRI quantitative analysis localised on the reference standard against FDG PET/CT to confirm reference standard positivity/negativity.

### Statistical analysis

All statistical analyses were performed using Prism (version 5.0; GraphPad Software, San Diego, CA). Distribution normality was assessed by the Kolmogorov–Smirnov test.

The Mann–Whitney test was used to assess difference of median ADC and normalised T2 signal intensity among (a) FDG PET/CT positive and negative lymph nodes with short-axis diameter ≥5 mm, (b) FDG PET/CT positive and negative nodes with 5- to 9-mm short-axis diameter, and (c) 5- to 9-mm short-axis diameter nodes and short-axis diameter ≥10-mm nodes. Interobserver (reader 1 vs. reader 2) and inter-sequence (STIR half-Fourier acquisition single-shot turbo spin echo vs. diffusion-weighted imaging) agreement of nodal size measurement as well as interobserver agreement for ADC and normalised T2 signal intensity were determined by Bland-Altman method [19].

We calculated ROC/AUC for classification of nodes (against the FDG PET/CT reference standard) for each reader for each quantitative parameter (DWI size, T2 size, normalised T2 signal intensity and ADC). The sensitivity and specificity for whole-body MRI nodal classification (for all nodes ≥5 mm) based on a size criteria short-axis diameter threshold of ≥10 mm indicating positivity were calculated for individual readers against the reference standard FDG PET/CT. We applied previously published ADC cut-off values (0.77, 1.15 and 1.79 ×10^−3^ mm^2^ s^−1^ corresponding to median (1.15×10^−3^ mm^2^ s^−1^ ), minimum (0.77×10^−3^ mm^2^ s^−1^ ) and maximum (1.79×10^−3^ mm^2^ s^−1^ ) ADC values for FDG PET/CT positive nodes) to determine classification performance for 5- to 9-mm “normal sized” lymph nodes. [20].

A short-axis diameter threshold of ≥10 mm was initially applied to all measured nodes, determining nodes ≥10 mm as positive. This was followed by application of ADC cut-offs to 5- to 9-mm short-axis diameter normal-size lymph nodes, ascribing nodes with ADC values below the threshold as positive. Sensitivity and specificity for nodal involvement based on combined short-axis diameter and apparent diffusion coefficient classification were determined for each of the three ADC thresholds.

## Results

Thirty-seven children and young adults (male/female: 16/21; mean age 16.1 years; range 12.8–18 years) all had required baseline and follow-up whole-body MRI sequences and were therefore eligible for inclusion in the study. Staging whole-body MRI was performed within a median of 3 days (range 0–19 days) of FDG PET/CT, without any complications and before treatment in all patients. Across the cohort, 3 children had Stage I, 11 had Stage II, 2 had Stage IIE, 8 had Stage III and 13 had stage IV disease. One hundred fifty-two nodal sites were positive on reference standard FDG PET/CT. Eighteen and 23 nodes were 5- to 9-mm short-axis diameter and positive according to reference standard FDG PET/CT for Reader 1 and Reader 2, respectively.

**Fig. 1:**
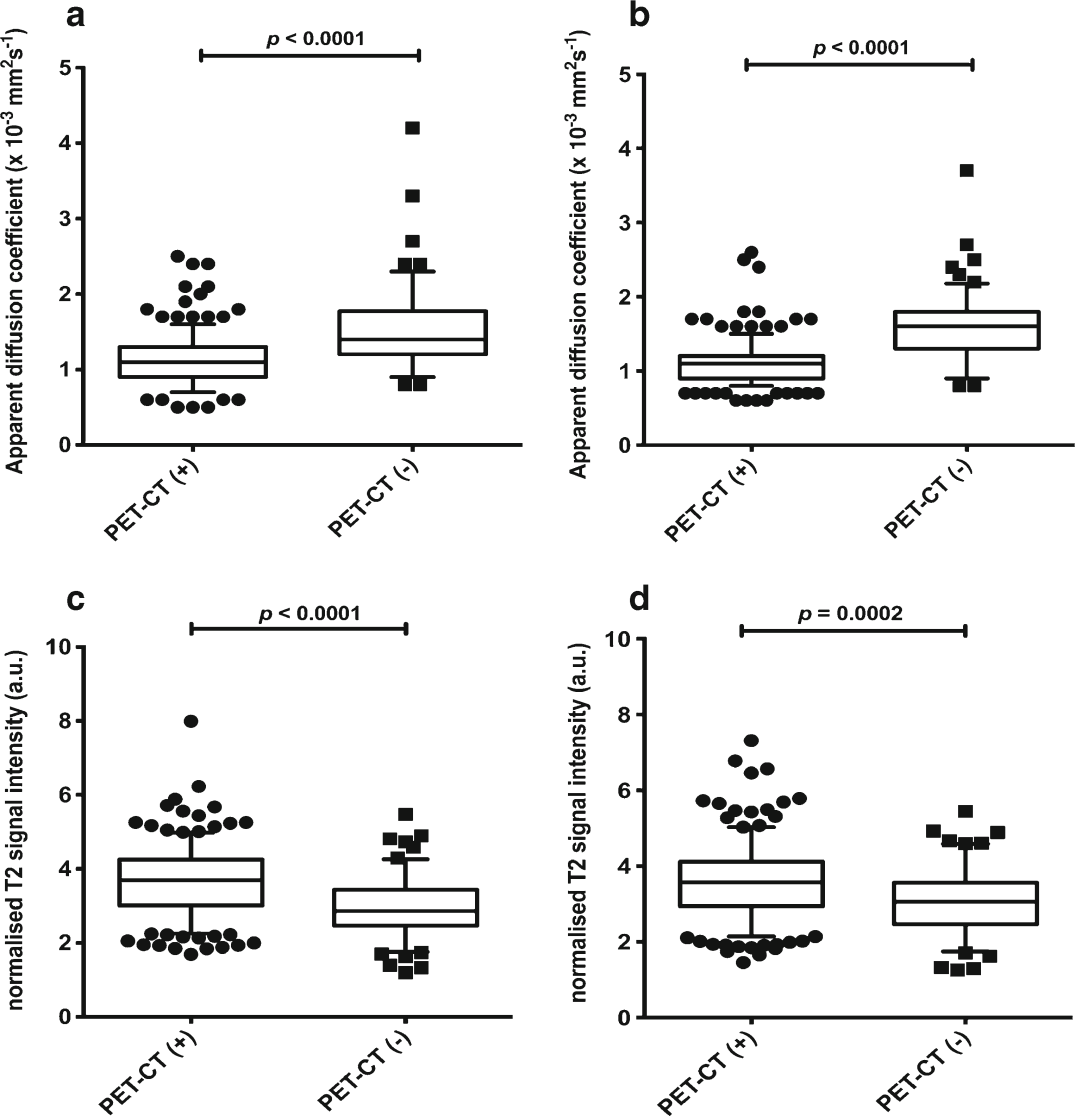
Box plots compare apparent diffusion coefficient (ADC) and normalised T2 signal intensity according to ^18^flourine-2-fluoro-2-deoxyglucose (FDG) positron emission tomography (PET)/CT positivity/negativity criteria for all nodes measuring 5 mm on whole-body MRI. Boxes represent 25th and 75th percentiles and the lines in the middle of the boxes represent the median values. Whiskers represent 10th and 90th percentiles and dots represent outliers. **a** Comparison of ADC measurements between FDG PET/CT-positive and -negative nodes for Reader 1 shows significantly lower values for FDG PET/CT-positive nodes compared to FDG PET/CT-negative nodes. **b** Comparison of ADC measurements between FDG PET/CT-positive and -negative nodes for Reader 2 shows significantly lower values for FDG PET/CT-positive nodes compared to FDG PET/CT-negative nodes. **c** Comparison of Normalised T2 signal intensity measurements between FDG PET/CT-positive and -negative nodes for Reader 1 shows significantly higher values for FDG PET/CT-positive nodes compared to FDG PET/CT-negative nodes. **d** Comparison of normalised T2 signal intensity measurements between FDG PET/CT-positive and -negative nodes for Reader 2 shows significantly higher values for FDG PET/CT-positive nodes compared to FDG PET/CT-negative nodes. Results are tabulated in Table 2. *a.u.* arbitrary unit

**Fig. 2:**
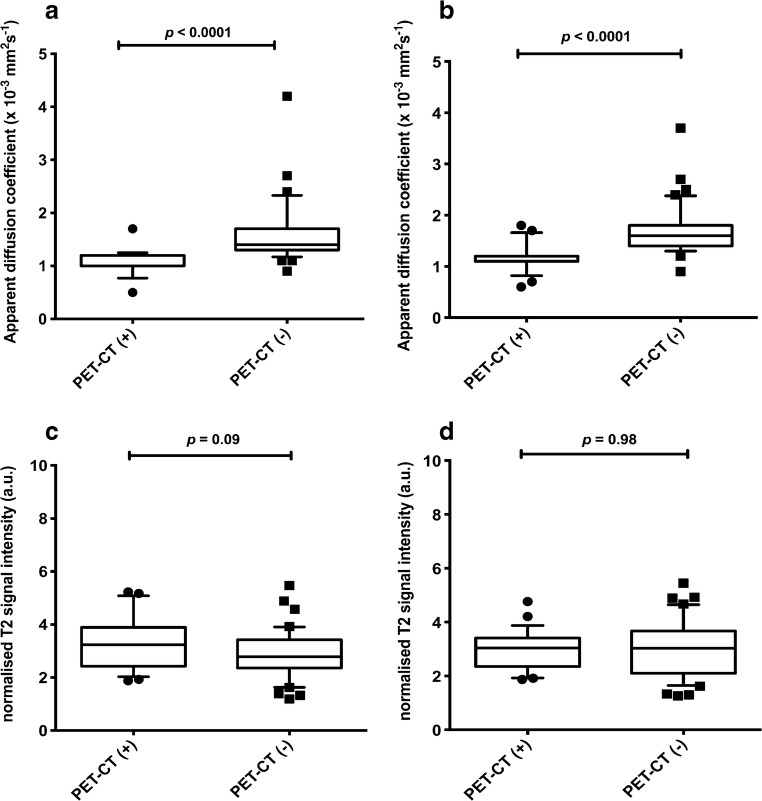
Box plots compare apparent diffusion coefficient (ADC) and normalised T2 signal intensity for nodes measuring 5–9 mm according to ^18^flourine-2-fluoro-2-deoxyglucose (FDG) positron emission tomography (PET)/CT positivity/negativity. Boxes represent 25th and 75th percentiles and the lines in the middle of the boxes represent the median values. Whiskers represent 10th and 90th percentiles and dots represent outliers. **a** Comparison of ADC measurements for 5- to 9-mm nodes for Reader 1 show significantly lower values for FDG PET/CT-positive nodes compared to FDG PET/CT-negative nodes. **b** Comparison of ADC measurements for 5- to 9-mm nodes for Reader 2 shows significantly lower values for FDG PET/CT-positive nodes compared to FDG PET/CT-negative nodes. **c** Comparison of Normalised T2 signal intensity measurements for 5- to 9-mm nodes for Reader 1 shows no significant difference for FDG PET/CT-positive nodes compared to FDG PET/CT-negative nodes. **d** Comparison of Normalised T2 signal intensity measurements for 5- to 9-mm nodes for Reader 2 shows no significant difference for FDG PET/CT-positive nodes compared to FDG PET/CT-negative nodes. Results are tabulated in Table 2. *a.u.* arbitrary unit

**Fig. 3:**
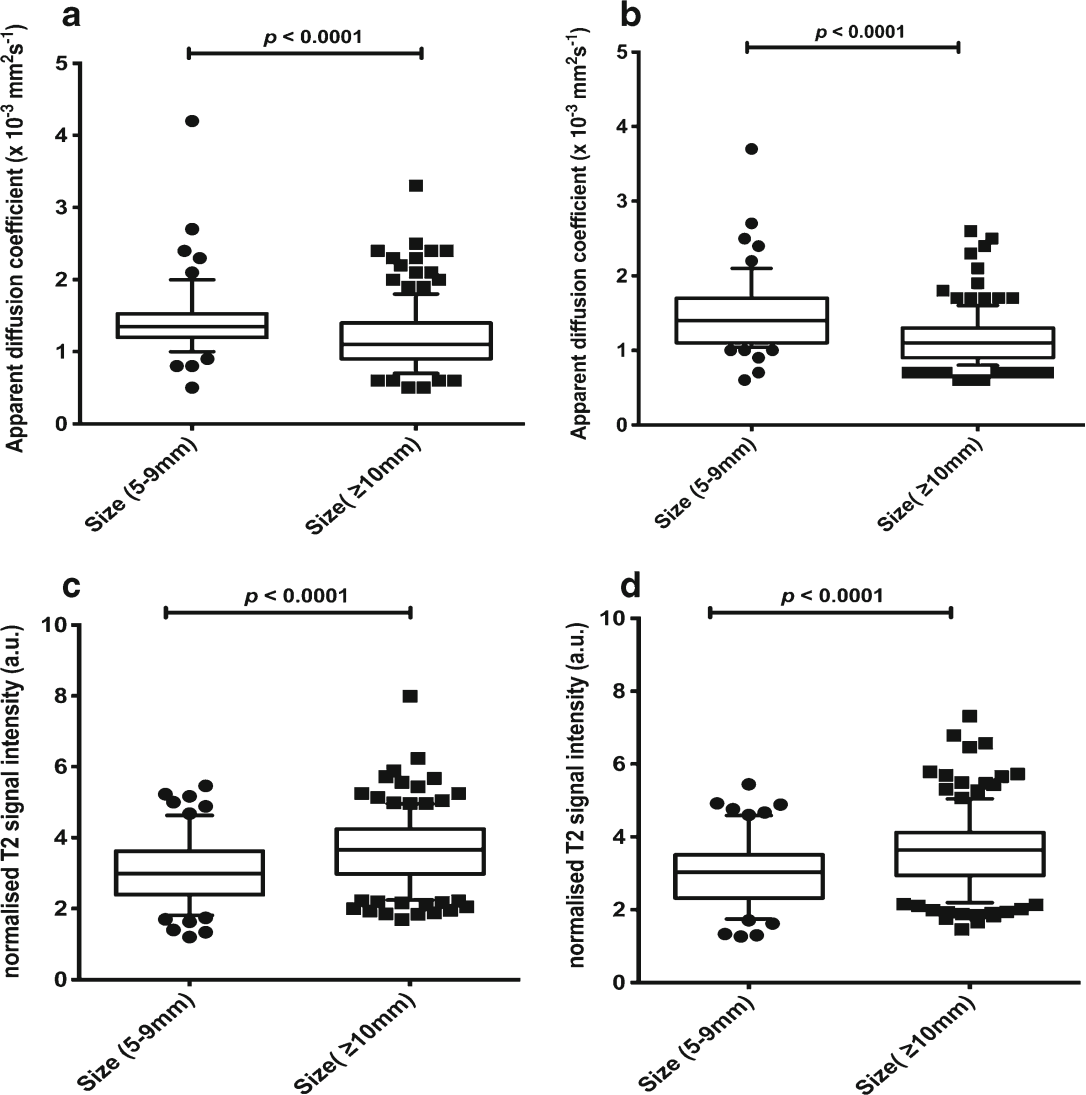
Box plots compare apparent diffusion coefficient (ADC) and normalised T2 signal intensity according to nodal short-axis-diameter measurements. Boxes represent 25th and 75th percentiles and the lines in the middle of the boxes represent the median values. Whiskers represent 10th and 90th percentiles and dots represent outliers. **a** Comparison of ADC measurements for Reader 1 shows significant difference between nodes measuring 5–9 mm and nodes measuring Size >10 mm. **b** Comparison of ADC measurements for Reader 2 shows significant difference between nodes measuring 5–9 mm and nodes measuring Size >10 mm. **c** Comparison of Normalised T2 signal intensity measurements for Reader 1 shows significant difference between nodes measuring 5–9 mm and nodes measuring Size >10 mm. **d** Comparison of Normalised T2 signal intensity measurements for Reader 2 shows significant difference between nodes measuring 5–9 mm and nodes measuring Size >10 mm. Results are tabulated in Table 2. *a.u.* arbitrary unit

**Fig. 4:**
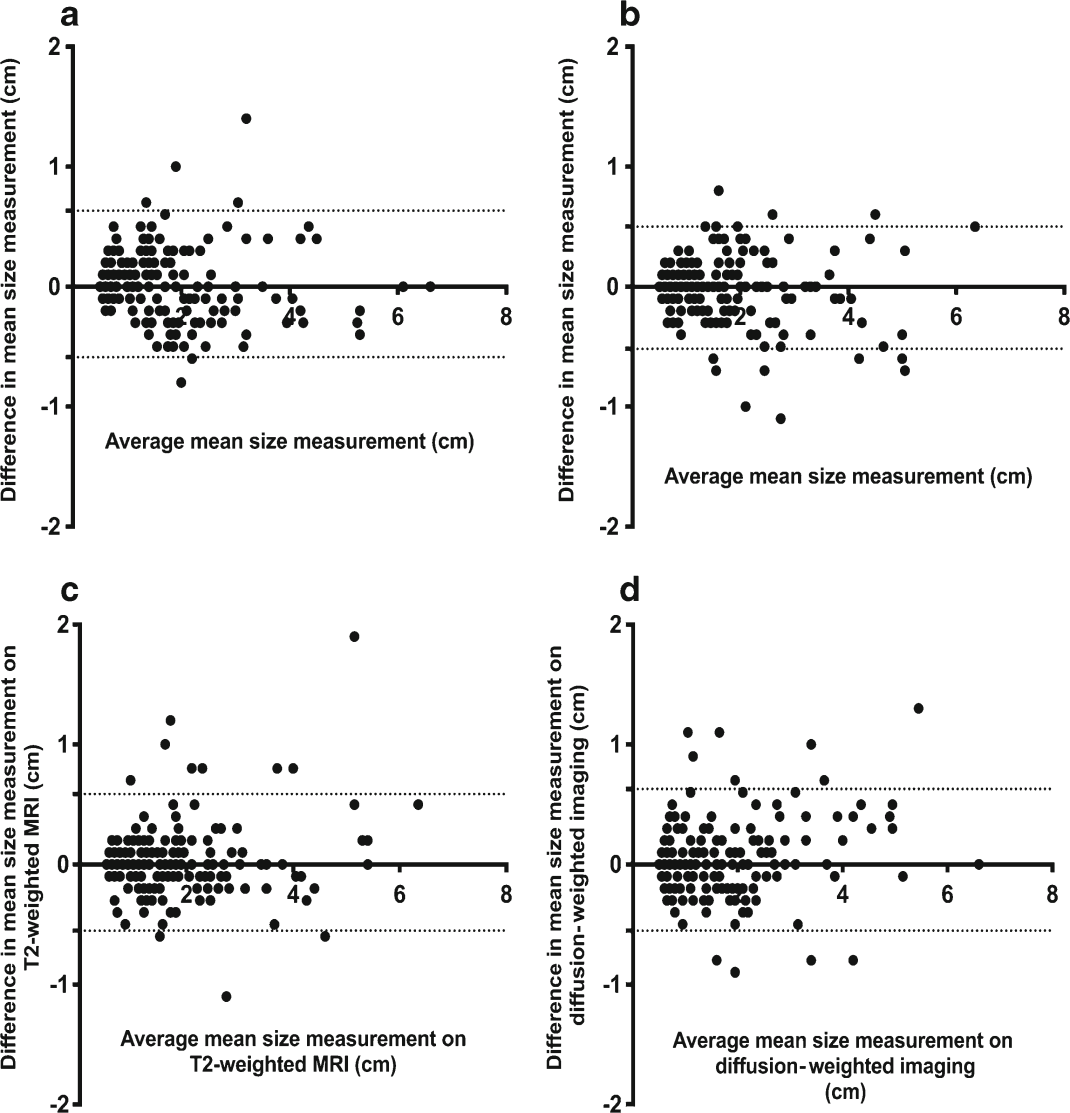
Bland-Altman plots for inter-sequence (axial short tau inversion recovery [STIR] half-Fourier-acquisition single-shot turbo spin echo vs. axial diffusion-weighted imaging [DWI; b=500 s/mm^2^] and interobserver (Reader 1 vs. Reader 2) agreements for nodal size measurement. Bias (*solid line*) and 95% limits of agreement (*dashed lines*) indicated. **a** For Reader 1, there was a bias of 0.020 cm and 95% limits of agreement of−0.59 to +0.63. **b** For Reader 2, there was a bias of −0.010 cm and 95% limits of agreement of−0.52 to +0.50 for inter-sequence size measurement agreements. **c** Short-axis nodal size measurements on axial STIR half-Fourier-acquisition single-shot turbo spin echo exhibit a bias of 0.017 cm and 95% limits of agreement of−0.55 to +0.58 between the two readers. **d** Short-axis nodal size measurements on axial DWI (b=500) exhibit a bias of 0.038 cm and 95% limits of agreement of−0.55 to +0.63 between the two readers

**Fig. 5:**
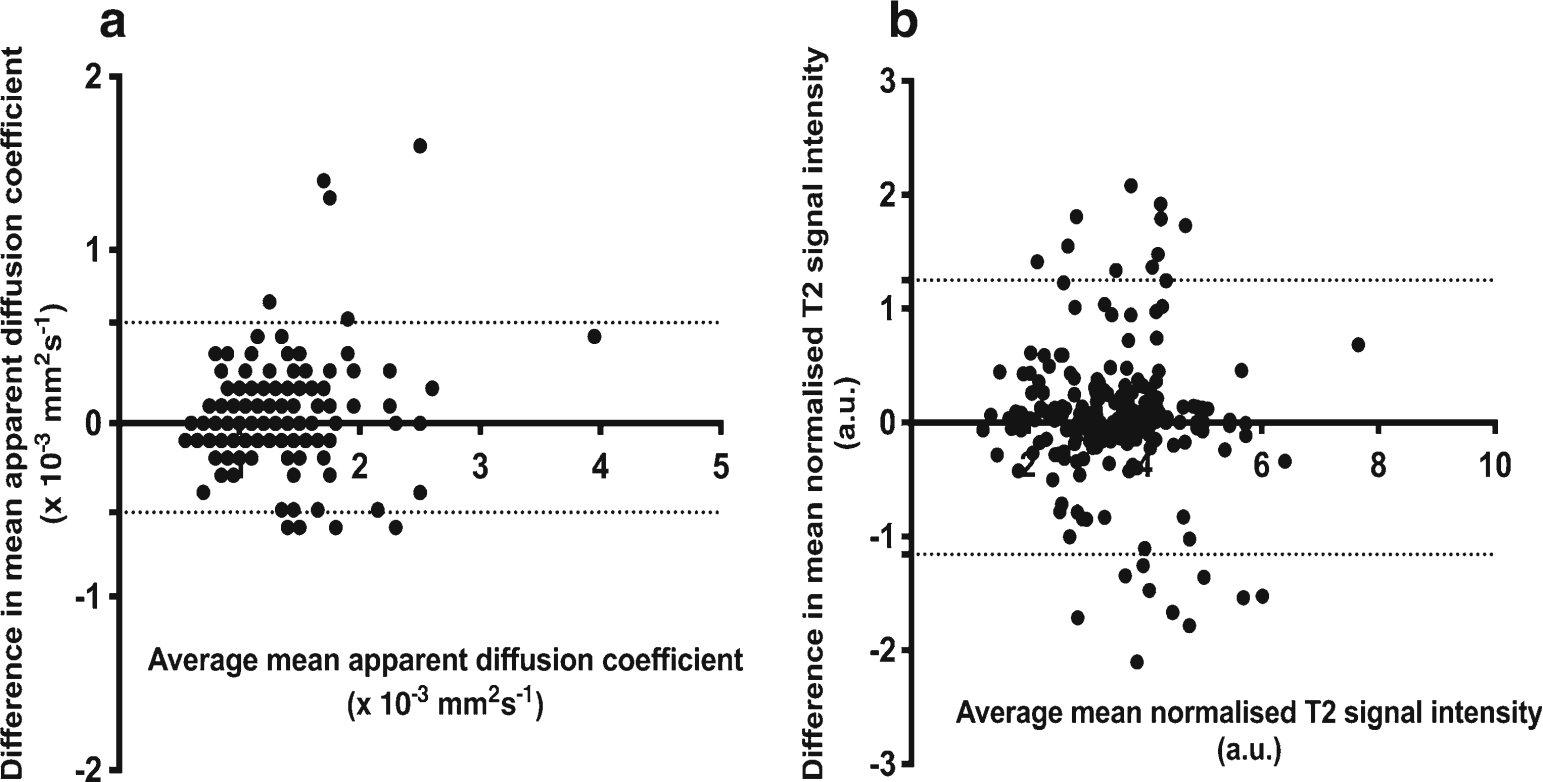
Bland-Altman plots for interobserver (Reader 1 vs. Reader 2) agreement of apparent diffusion coefficient (ADC) and normalised T2-signal-intensity measurements for lymph nodes with short-axis diameter ≥5 mm. Bias (*solid line*) and 95% limits of agreement (*dashed lines*) indicated. **a** ADC measurements exhibit a bias of 0.03×10^−3^ mm^2^ s^−1^ and 95% level of agreement of−0.52 to +0.58. **b** Normalised T2 signal intensity measurements show a bias of 0.04 (a.u.) and 95% level of agreement of−1.16 to +1.25. *a.u.* arbitrary unit

Based on the Kolmogorov–Smirnov test, both ADC and normalised T2-signal-intensity metrics were not normally distributed. Median ADC and normalised T2 signal intensity for FDG PET/CT-positive and FDG PET/CT-negative lymph nodes are shown in Fig. 1 and Table 2 for both radiologists. For lymph nodes with 5- to 9-mm short-axis diameter, median ADC was significantly lower (*P*<0.0001) for FDG PET/CT-positive compared with FDG PET/CT-negative lymph nodes, whilst there was no significant difference in median normalised T2 signal intensity (Fig. 2, Table 2). Median ADC was significantly higher and median normalised T2 signal intensity significantly lower in nodes with 5- to 9-mm short-axis diameter compared with nodes with ≥10-mm short-axis-diameter (Fig. 3 and Table 2). Bland-Altman plots for interobserver and inter-sequence size measurement agreements (DWI b=500 s/mm^2^ and STIR half-Fourier-acquisition single-shot turbo spin echo) are shown in Fig. 4. Bland-Altman plots of interobserver agreements for ADC and normalised T2 signal intensity measurements are represented in Fig. 5.

**Table 2:** Comparison of median apparent diffusion coefficient and normalised T2 signal intensity of lymph nodes based on: (1) ^18^flourine-2-fluoro-2-deoxyglucose (FDG) positron emission tomography (PET)/CT positivity/negativity criteria (Analysis 1); (2) short-axis diameter (Analysis 2); and (3) for 5- to 9-mm nodes, FDG PET/CT positivity/negativity criteria (Analysis 3)

	Median apparent diffusion coefficient (× 10^−3^ mm^2^ s^−1^)		Median normalised T2 signal intensity (a.u.)	
	Reader 1	Reader 2	Reader 1	Reader 2
Analysis 1				
PET/CT (+)	1.10 (interquartile range 0.40) [number=152]	1.10 (interquartile range 0.30) [number=152]	3.70 (interquartile range 1.24) [number=152]	3.58 (interquartile range 1.18) [number=152]
PET/CT (-)	1.40 (interquartile range 0.57) [number=60]	1.60 (interquartile range 0.50) [number=61]	2.86 (interquartile range 0.97) [number = 60]	3.05 (interquartile range 1.11) [number=61]
*P*-value^a^	**<0.0001**	**<0.0001**	**<0.0001**	**0.0002**
Analysis 2				
Nodes (5–9 mm)	1.35 (interquartile range 0.32) [number=54]	1.40 (interquartile range 0.60) [number=63]	2.98 (interquartile range 1.23) [number=64]	3.03 (interquartile range 1.19) [number=68]
Nodes (≥10 mm)	1.10 (interquartile range 0.50) [number=158]	1.10 (interquartile range 0.40) [number=150]	3.66 (interquartile range 1.27) [number=148]	3.64 (interquartile range 1.18) [number=145]
*P*-value^a^	**<0.0001**	**<0.0001**	**<0.0001**	**<0.0001**
Analysis 3				
5–9-mm nodes and PET/CT (+)	1.20 (interquartile range 0.20) [number=18]	1.10 (interquartile range 0.10) [number=23]	3.24 (interquartile range 1.46) [number=18]	3.04 (interquartile range 1.06) [number=23]
5- to 9-mm nodes and PET/CT (-)	1.40 (interquartile range 0.40) [number=36]	1.60 (interquartile range 0.40) [number=40]	2.78 (interquartile range 1.06) [number=36]	3.03 (interquartile range 1.57) [number=40]
*P*-value^a^	**<0.0001**	**<0.0001**	0.09	0.98

*a.u.* arbitrary unit

^a^Mann–Whitney test; *P*<0.05 is significant (bold)

### Receiver operating characteristic/area under the curve analysis (all nodes)

**Fig. 6:**
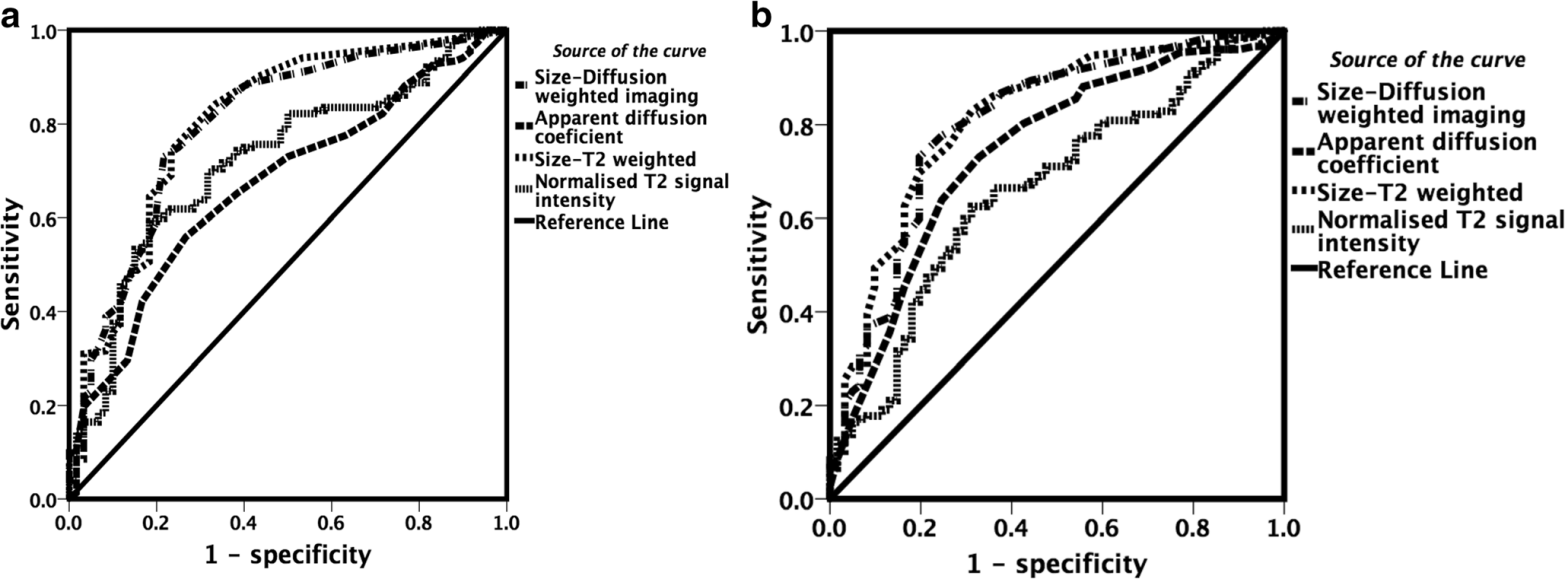
Receiver operating characteristic (ROC)/area under the curve (AUC) analysis for classification of nodes for each quantitative parameter measurement made by (**a**) Reader 1, and (**b**) Reader 2. Size measurements on diffusion-weighted imaging (DWI) and short tau inversion recovery (STIR) half-Fourier-acquisition single-shot turbo-spin-echo images achieved the highest AUC for both readers: 0.80, 95% confidence interval [CI] = 0.73–0.87, and 0.81, 95% CI=0.75–0.88 for Reader 1; and 0.80, 95% CI=0.73–0.87, and 0.81, 95% CI=0.74–0.87 for Reader 2, respectively. ROC/AUC for the apparent diffusion coefficient was 0.74 (95% CI=0.67–0.82) and 0.67 (95% CI=0.59–0.75) for Readers 1 and 2, respectively. ROC/AUC for normalised T2 signal intensity was 0.66 (95% CI=0.58–0.74) and 0.72 (95% CI=0.64–0.79) for Readers 1 and 2, respectively

ROC/AUC graphs are presented in Fig. 6. For Reader 1, the AUCs for nodal size measurement on b=500 s/mm^2^ DWI and for STIR half-Fourier-acquisition single-shot turbo spin echo were 0.80 (95% confidence interval [CI] = 0.73–0.87) and 0.81 (95% CI=0.75–0.88), respectively. For Reader 2, the AUCs for nodal size measurement on b=500 s/mm^2^ DWI and for STIR half-Fourier acquisition single-shot turbo spin echo were 0.80 (95% CI=0.73–0.87) and 0.81 (95% CI=0.74–0.87), respectively. The AUCs for the apparent diffusion coefficient and for the normalised T2 signal intensity for Reader 1 were 0.74 (95% CI=0.67–0.82) and 0.66 (95% CI=0.58–0.74), respectively. For Reader 2 the ADC and the normalised T2 signal intensity AUCs were 0.67 (95% CI=0.59–0.75) and 0.72 (95% CI=0.64–0.79), respectively.

### Short-axis-diameter threshold-based sensitivity/specificity (all nodes)

The sensitivity and specificity for short-axis-diameter ≥10-mm size threshold as applied to all lymph nodes were 84.2% and 66.7% for Reader 1 and 82.9% and 68.9% for Reader 2.

### Apparent diffusion coefficient threshold sensitivity/specificity (for nodes 5–9 mm)

The sensitivity and specificity for the ADC cut-off values of 0.77, 1.15 and 1.79×10^−3^ mm^2^ s^−1^ as applied to 5- to 9-mm short-axis-diameter lymph nodes is tabulated in Table 3.

**Table 3:** Sensitivity and specificity of apparent diffusion coefficient (ADC) for classification of disease in 5- to 9-mm short-axis-diameter lymph nodes

	Sensitivity		Specificity	
ADC values	Reader 1	Reader 2	Reader 1	Reader 2
0.77×10^−3^ mm^2^ s^−1^	5.6%	8.7%	100%	100%
1.15×10^−3^ mm^2^ s^−1^	33.3%	63.6%	92%	94.4%
1.79×10^−3^ mm^2^ s^−1^	100%	92%	24%	35.3%

### Combined short-axis-diameter and apparent diffusion coefficient thresholds sensitivity/specificity (all nodes)

**Fig. 7:**
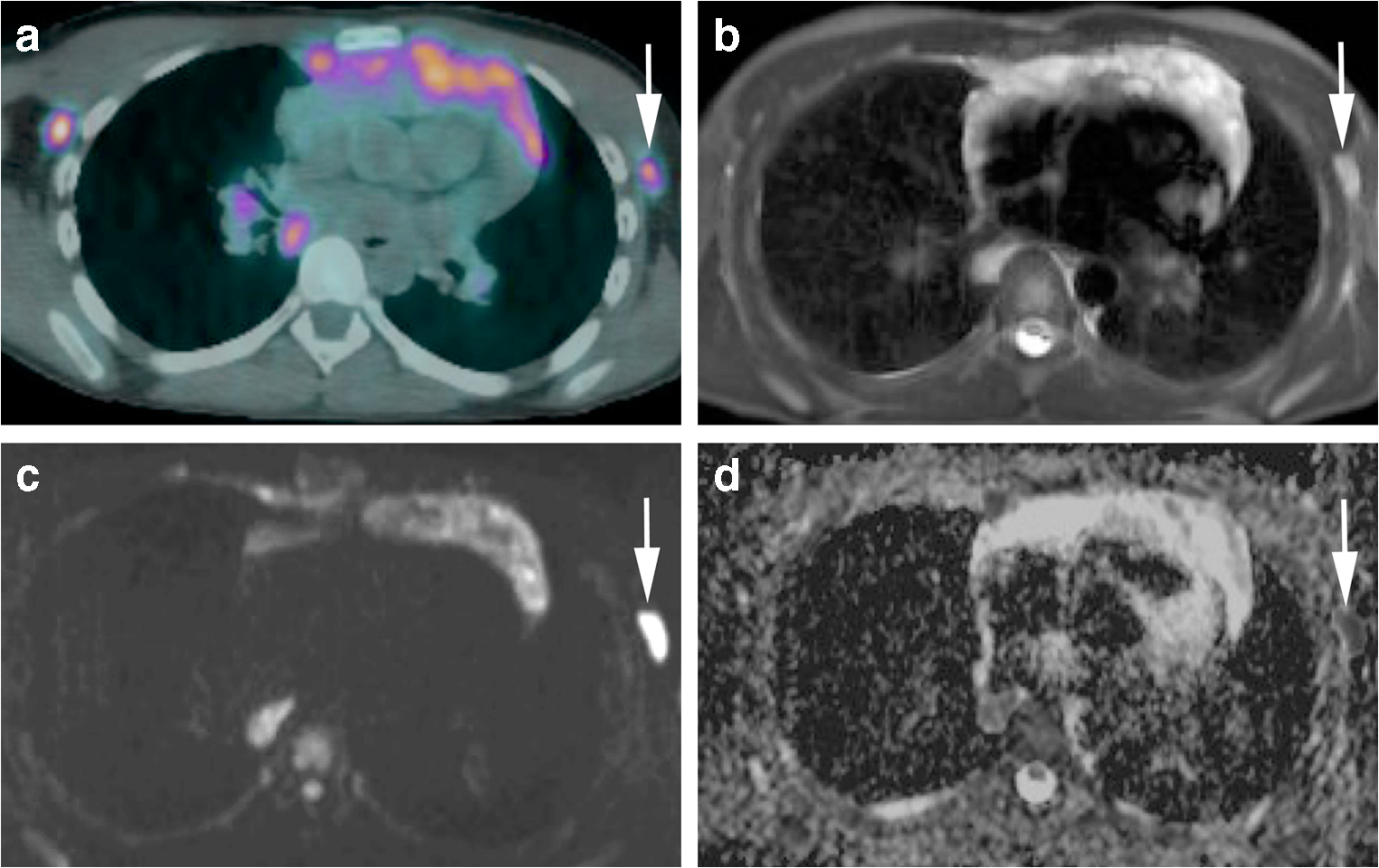
Images in a 15-year-old boy with Hodgkin lymphoma. **a** Axial ^18^flourine-2-fluoro-2-deoxyglucose (FDG) positron emission tomography (PET)/CT shows a positive left axillary node (*arrow*). **b** Axial short tau inversion recovery half-Fourier acquisition single-shot turbo-spin-echo (repetition time/echo time [TR/TE] = 800/60 ms) MR image demonstrates an 8-mm short-axis-diameter left axillary node (*arrow*). **c** Axial diffusion-weighted imaging (DWI; b=500 s/mm^2^; TR/TE=4,900/66 ms) demonstrates an 8-mm short-axis-diameter left axillary node. These findings would result in false-negative nodal classification according to the threshold of short-axis diameter ≥10 mm. **d** Apparent diffusion coefficient (ADC) map demonstrates the same left axillary node (*arrow*) with an ADC of 0.8×10^−3^ mm^2^ s^−1^. The node was deemed positive on whole-body MRI using the combined short-axis diameter ≥10 mm and ADC cut-off of <1.15×10^−3^ mm^2^ s^−1^ classification — a true-positive result compared with using the FDG PET/CT reference

**Fig. 8:**
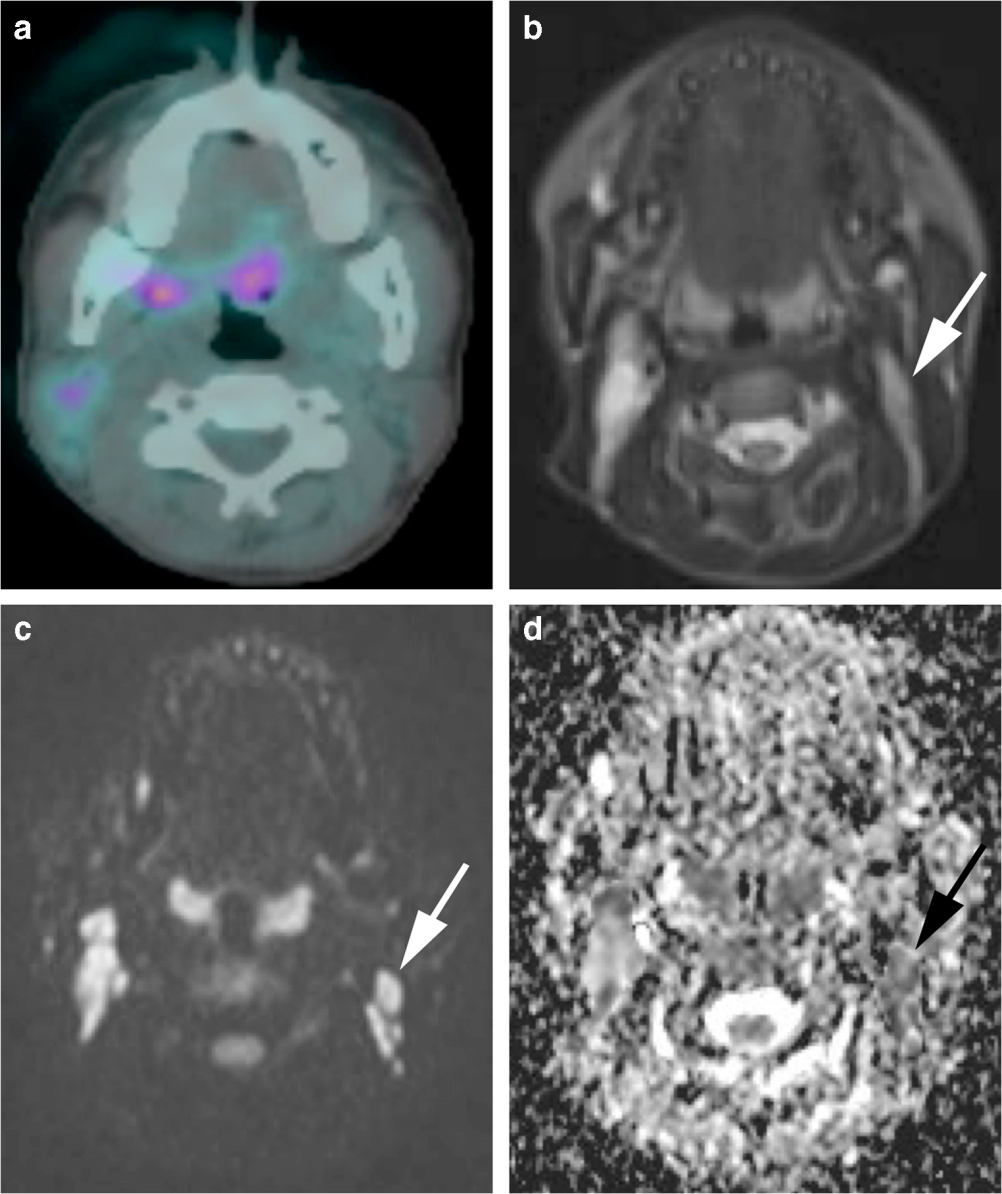
Images in a 16-year-old girl with Hodgkin lymphoma. **a**^18^Flourine-2-fluoro-2-deoxyglucose (FDG) positron emission tomography (PET)/CT is deemed negative for left cervical nodal involvement. **b** Axial short tau inversion recovery half-Fourier-acquisition single-shot turbo-spin-echo (repetition time/echo time [TR/TE] = 800/60 ms) MR image demonstrates a 5-mm short-axis-diameter left cervical node (*arrow*). **c** Axial diffusion-weighted imaging (DWI; b=500 s/mm^2^; TR/TE=4,900/66 ms) demonstrates a 5-mm short-axis-diameter left cervical node (*arrow*). These findings would result in true-negative nodal classification. **d** Apparent diffusion coefficient (ADC) map demonstrates the same left cervical node (*arrow*) with ADC of 0.9×10^−3^ mm^2^ s^−1^. The node was deemed positive on whole-body MRI by combined short-axis diameter ≥10 mm and ADC cut-off value of <1.15×10^−3^ mm^2^ s^−1^ classification — a false-positive result compared with using the FDG PET/CT reference

The sensitivity and specificity of combining short-axis-diameter ≥10-mm size threshold applied to all nodes, followed by ADC cut-off values of 0.77, 1.15 and 1.79×10^−3^ mm^2^ s^−1^ to 5- to 9-mm lymph nodes for both readers, are given in Table 4. Figures 7 and 8 demonstrate examples of true-positive and false-positive whole-body MRI nodal classification using the combined short-axis diameter and ADC threshold.

**Table 4:** Sensitivity and specificity of combined short-axis-diameter ≥10-mm size threshold applied to all nodes, followed by apparent diffusion coefficient (ADC) cut-off values applied to 5- to 9-mm short-axis-diameter lymph nodes

	Sensitivity		Specificity	
ADC values	Reader 1	Reader 2	Reader 1	Reader 2
0.77×10^−3^ mm^2^ s^−1^	88.8%	86.1%	60.0%	67.2%
1.15×10^−3^ mm^2^ s^−1^	92.1%	95.3%	56.7%	65.6%
1.79×10^−3^ mm^2^ s^−1^	100%	100%	16.7%	19.7%

## Discussion

Whole-body MRI is being increasingly advocated as an alternative or adjunct to FDG PET/CT for staging and response monitoring in paediatric lymphoma. Previously published works have shown that morphological MRI and DWI can be implemented in whole-body MRI protocols for assessing the extent of disease [9, 13]. MRI signal itself can be made sensitive to the presence of pathology, and as such, MRI offers the opportunity to use signal characteristics as well as morphological features to classify disease.

Size measurement is conventionally used to determine nodal positivity. We investigated whether the performance of whole-body MRI for nodal classification, based on size measurement, was different when measurements were made on whole-body DWI rather than anatomical whole-body STIR half-Fourier-acquisition single-shot turbo-spin-echo imaging. We found that measurements made on either sequence yielded a comparable diagnostic accuracy for detecting involved lymph nodes and that size measurement provides the best classifier for nodal disease status.

In our study, diseased lymph nodes had significantly lower ADC values than benign lymph nodes (as highlighted by the FDG PET/CT reference standard); this finding supported the results of prior investigators [21, 22]. For instance, Perrone et al. [23] demonstrated that the mean ADC value for malignant cervical lymph nodes was significantly lower than that of benign lymphadenopathy (0.85×10^−3^ mm^2^ s^−1^ and 1.44×10^−3^ mm^2^ s^−1^, respectively, *P*<0.01). In another study, the authors showed that compared to benign lymphadenopathy and metastatic lymphadenopathy from head and neck cancers, lymphomatous lymph nodes had a significantly lower mean ADC value [21]. Whilst our results demonstrate good classification by ADC value (ROC/AUC of 0.67–0.74), we note that the performance of the ADC was not greater than simply measuring nodal size (ROC/AUC 0.80–0.81).

We also noticed that for nodes deemed negative for disease based on size criteria (measuring 5–9 mm) there was significant difference in ADC between disease-positive and disease-negative nodes (*P*<0.0001). It is not surprising, therefore, that after applying ADC cut-off values [20] to 5- to 9-mm lymph nodes, there was an overall increase in whole-body DWI’s sensitivity for both readers. However, the increase in whole-body DWI’s sensitivity came at the expense of noticeable decrease in specificity. We conclude that early nodal involvement (where size remains <1 cm) cannot be reliably classified using DWI and hypothesise that this likely reflects insufficient increase in cellularity to significantly alter water diffusion [24].

Our result for interobserver reproducibility of ADC measurements for lymph nodes in children and adolescents with Hodgkin lymphoma emulates previous findings for nodal ADC reproducibility in healthy volunteers [25, 26]. Moreau et al. [26] found that the inter-reader reproducibility for their ADC measurements showed an absolute bias of 0.045×10^−3^ mm^2^ s^−1^ (level of agreement −0.146; 0.056). Although both studies used a different protocol compared to our study, they highlighted that ADC measurement in healthy volunteers might not always be adequately reproducible and that a reliable use of ADC values requires further technical advances and systematic quality control [25, 26].

Short tau inversion recovery half-Fourier-acquisition single-shot turbo-spin-echo images generate contrast to highlight water whilst nulling signal from fat [27]. In theory, replacement of the normal fatty hilum by cellular infiltrate as occurs in pathology should increase the STIR half-Fourier-acquisition single-shot turbo-spin-echo signal from diseased lymph nodes [28]. This was reflected in our results, with FDG PET/CT-positive lymph nodes demonstrating approximately 20% increased signal on STIR half-Fourier-acquisition single-shot turbo-spin-echo images compared to FDG PET/CT-negative nodes. As a univariate classification parameter, normalised T2 signal-intensity classification performance (ROC/AUC 0.66–0.72) was not as high as ADC or nodal size measurement parameters.

Ohno et al. [29] evaluated 135 metastatic and 135 non-metastatic mediastinal and hilar lymph nodes in their cohort of 93 patients with N1-N3 non-small-cell lung cancer using 1.5-T STIR turbo-spin-echo MRI, diffusion-weighted imaging and FDG PET/CT, with the reference standard being histopathological samples. In line with our results, they showed that lymph node signal intensity as normalised to muscle signal intensity (lymph-node-to-muscle ratio) on STIR turbo-spin-echo sequence was significantly higher in metastatic lymph nodes compared to non-metastatic lymph nodes (mean lymph-node-to-muscle ratio 1.5 ± 0.3 [range 0.6–2.2] and 1.0 ± 0.3 [range 0.4–1.8] for metastatic and non-metastatic lymph nodes, respectively, *P*<0.0001). However contrary to our results, the performance of lymph-node-to-muscle ratio for determining metastatic lymph nodes was higher than that of ADC values in their cohort. This might be caused by two major differences between the two studies: first, the underlying malignancies were different between the two cohorts; second, Ohno et al. [29] only investigated mediastinal and hilar lymph nodes whilst we included all the measurable nodal stations above and below the diaphragm.

In our study, we included the nodal stations comprising only measurable nodes with short-axis diameter ≥5 mm, for two reasons. First, quantitative analysis of small nodes is more likely to be affected by partial volume errors compared with larger nodes. Second, and more important, almost all <5-mm lymph nodes in paediatric patients with Hodgkin lymphoma are negative on FDG PET/CT (indeed, we did not find a single FDG PET/CT-positive node in our cohort) and do not present a diagnostic challenge. Application of quantitative metrics should be for nodes in which there might be a diagnostic dilemma. Studies that include large numbers of nodes <5 mm within their population are likely to demonstrate artificially high levels of specificity for the technique being assessed.

Our results have direct clinical implications. They suggest that ADC values should not be used as a discriminator of nodal disease status in children with lymphoma and confirm the dominance of nodal size as the best classifier of disease status. Furthermore, if diffusion weighted imaging is used for staging paediatric lymphoma, it is sufficient to make measurements of nodal size directly from diffusion-weighted images (b=500 s/mm^2^) rather than from anatomical imaging such as STIR half-Fourier-acquisition single-shot turbo-spin-echo images. This might help to reduce scan times by providing an opportunity to replace STIR half-Fourier-acquisition single-shot turbo spin echo with a faster anatomical acquisition such as modified DIXON imaging [30, 31] or obviating the requirement for DWI based on no additional classification value. Limiting overall scan time is particularly important for paediatric patients and has direct heath care cost implications.

There were limitations to our study. First, we did not have histopathological confirmation for positivity/negativity for nodal involvement by lymphoma. We used FDG PET/CT as the reference standard because this is the current gold standard modality of imaging with excellent performance [32]. We have assumed that discordances between whole-body MRI and the FDG PET/CT reference are related to limitations in sensitivity/specificity of the whole-body MRI technique, but we acknowledge that a small number of lymph nodes might also be misclassified by FDG PET/CT. Although ethically and technically challenging, a prospective study with histological sampling to resolve discrepancies would further aid comparison between techniques. A second limitation of the study is the generalisability of whole-body MRI quantitative features across institutions. For example, absolute ADC values are known to be dependent on MRI protocol parameters and scanner platform [33, 34]. However our best-performing classifier remained nodal size, and classification was not significantly improved by ADC values. Third, we used the highest b value of 500 s/mm^2^ for DWI disease assessment. We acknowledge that a higher b value of 800–1,000 s/mm^2^ would have been in line with current recommendations [35]; however the ADC cut-off values were derived from previous work using a similar DWI protocol to the current study [19]. Finally, whilst we excluded nodes with short-axis diameter <5 mm from quantitative analysis to minimise the partial voluming effect, partial voluming effect on our quantitative measurements cannot be entirely excluded.

## Conclusion

We have demonstrated that both whole-body DWI and whole-body STIR half-Fourier-acquisition single-shot turbo-spin-echo MRI size measurements had similarly very good performance for classifying nodal disease involvement in children with known lymphoma. Combined short-axis diameter and ADC thresholds marginally improve sensitivity and drop specificity compared with size classification alone. We continue to advocate the use of anatomical criteria for nodal disease classification and raise caution over the interpretation of ADC value as a classifier of disease, particularly in normal-size (5- to 9-mm short-axis diameter) lymph nodes.
